# The anti-influenza virus drug, arbidol is an efficient inhibitor of SARS-CoV-2 in vitro

**DOI:** 10.1038/s41421-020-0169-8

**Published:** 2020-05-02

**Authors:** Xi Wang, Ruiyuan Cao, Huanyu Zhang, Jia Liu, Mingyue Xu, Hengrui Hu, Yufeng Li, Lei Zhao, Wei Li, Xiulian Sun, Xinglou Yang, Zhengli Shi, Fei Deng, Zhihong Hu, Wu Zhong, Manli Wang

**Affiliations:** 1 State Key Laboratory of Virology, Wuhan Institute of Virology, Center for Biosafety Mega-Science, Chinese Academy of Sciences, Wuhan, 430071 China; 2 National Engineering Research Center for the Emergency Drug, Beijing Institute of Pharmacology and Toxicology, Beijing, 100850 China; 3 University of the Chinese Academy of Sciences, Beijing, 100049 China; 4 National Virus Resource Center, Wuhan Institute of Virology, Chinese Academy of Sciences, Wuhan, 430071 China

**Keywords:** Cell biology, Molecular biology

Dear editor,

Since December 2019, a novel disease COVID-19 caused by severe acute respiratory syndrome coronavirus 2 (SARS-CoV-2) rapidly spread to over 200 countries and infected over 1.50 million people including 92,798 deaths (data as of April 10, 2020). On March 11, the World Health Organization (WHO) characterized COVID-19 as a pandemic, and called for accelerating diagnostics, vaccines, and drugs developments to combat this novel disease. Apart of the new coronavirus, influenza virus infections have been a consistent threat to the global public health over the years. In the United States alone, the Centers for Disease Control and Prevention (CDC) estimates that, so far during the 2019–2020 winter season, there have been at least 39 million illnesses, 400,000 hospitalizations and 24,000 deaths from influenza (https://www.cdc.gov/flu/weekly/index.htm). Considering the current concomitant circulation of SARS-CoV-2 and influenza virus infections, the exploration of available and viable anti-influenza drugs to treat both diseases is of great interest.

Actually, in the early stages of the outbreak of COVID-19, some anti-flu drugs (for example, oseltamivir) have been applied for the treatment of COVID-19 patients^1,2^. Previously, we reported that favipiravir (T705), an anti-influenza drug approved in Japan and China, showed a certain efficacy against SARS-CoV-2 in vitro^3^. In addition, arbidol, an anti-influenza drug targeting the viral hemagglutinin (HA) is being used in a clinical trial against COVID-19 (ChiCTR2000029573) and has been recently added to the Guidelines for the Diagnosis and Treatment of COVID-19 (sixth and seventh editions) in China. A recent retrospective study suggested that arbidol treatment showed tendency to improve the discharging rate and decrease the mortality rate of COVID-19 patients^4^. However, to our knowledge, there has been no systematical analysis about the efficacy of anti-influenza drugs against SARS-CoV-2.

In this study, we evaluated six currently available and licensed anti-influenza drugs against SARS-CoV-2. The drugs include arbidol, baloxavir, laninamivir, oseltamivir, peramivir, and zanamivir^5,6^. The M2 inhibitors (amantadine and rimantadine) were not considered in this study since they were not recommended for treating influenza by WHO due to drug resistance. First, the cytotoxicity of the compounds in African green monkey kidney cells, Vero E6 (ATCC-1586) was measured by a standard cell counting kit-8 (CCK8) assay. Then, the cells were infected with SARS-CoV-2 at a multiplicity of infection (MOI) of 0.05 in the presence of either compound or dimethyl sulfoxide (DMSO) control. The dose–response curves were determined by quantification of viral RNA copy numbers in the supernatant of infected cell at 48 h post infection (p.i.). As demonstrated in Fig. 1a, arbidol efficiently inhibited virus infection in vitro. The 50% maximal effective concentration (EC_50_) and the 50% cytotoxic concentration (CC_50_) of arbidol was 4.11 (3.55–4.73) and 31.79 (29.89–33.81) μM, respectively, and the selectivity index (SI = CC_50_/EC_50_) was 7.73. Baloxavir partially inhibited SARS-CoV-2 infection (~29%) at a high concentration of 50 μM (Fig. 1a). In contrast, laninamivir, oseltamivir, peramivir, and zanamivir did not exhibit anti-SARS-CoV-2 activity even at the highest drug concentrations (Fig. 1a). The antiviral effect of the compounds was also evaluated by observing cytopathic effects (CPE) and immunofluorescence staining of infected cells. As shown in Supplementary Fig. S1, at 48 h p.i. only in cells treated with arbidol, but not with the other five drugs, viral NP expression and CPE due to SARS-CoV-2 was substantially reduced. To be noted, we also tried some human lung cell lines, for example human embryo lung fibroblasts MRC-5 and lung cancer cell line Calu-3, however, they were not very efficient for SARS-CoV-2 replication, and therefore were not used for this study.

**Fig. 1:**
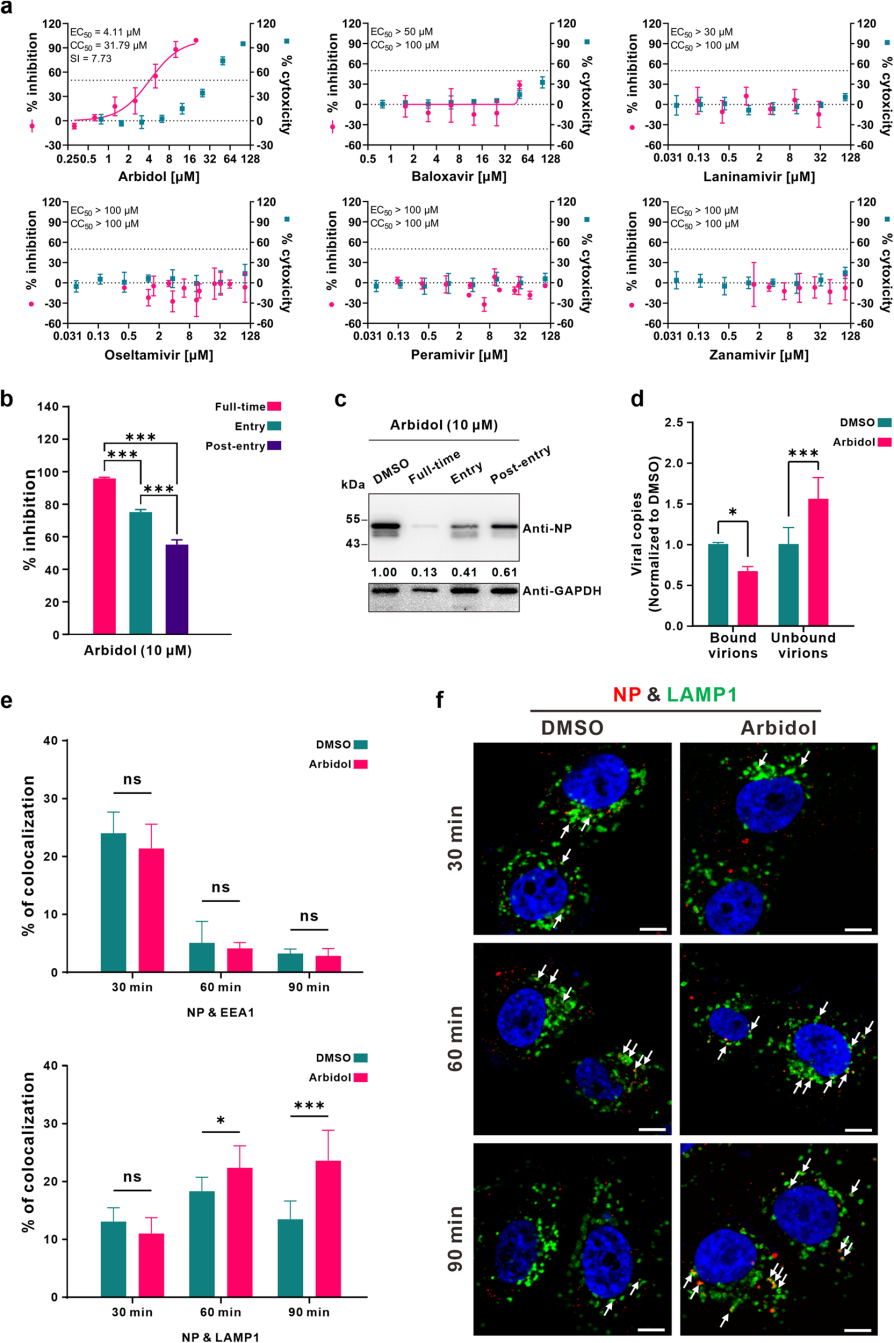
Comparative antiviral efficacy of anti-influenza drugs and the mode of actions of arbidol against SARS-CoV-2 infection in vitro. **a** Antiviral activities of the drugs. The antiviral efficacy was evaluated in Vero E6 cells by qRT-PCR analysis of virus yield at 48 h p.i. Data represent the mean ± standard deviation (SD) from two independent repeats. **b**, **c** Time-of-addition experiment of arbidol. Three experimental groups (Full-time, Entry, and Post-entry) were set up as described in the Supplementary Methods. At 16 h p.i., virus yield in the cell supernatant was quantified by qRT-PCR (**b**), and the expression of NP in infected cells was analyzed by western blots (**c**). The values below the blot represent the relative band intensity (NP/GAPDH) normalized to that of the DMSO group. **d** Impact of arbidol on SARS-CoV-2 binding. Vero E6 cells were treated with arbidol (10 μM) or DMSO for 1 h prior to infection with SARS-CoV-2 at 4 °C for 1 h. The supernatant (unbound virions) and the cells containing bound virions (bound virions) were collected for quantification of viral RNA copies by qRT-PCR. **e**, **f** Effect of arbidol on intracellular trafficking of SARS-CoV-2. The co-localization of virions with EEs or LEs was analyzed by immunofluorescence assays as described in the Supplementary Methods. **e** The portion of virions that co-localized with EEs or ELs in each group (*n* > 150 cells) was quantified by Image J. **f** Representative confocal microscopic images of virions (red) and LAMP1^+^ ELs (green) in each group. The nuclei (blue) were stained with Hoechst 33258 dye. White arrows: virions co-localized with ELs; bars: 10 μm. For (**b**) and (**e**), statistical analysis was performed using a one-way analysis of variance (ANOVA) with GraphPad Prism. For (**d**), statistical analysis was performed and calculated by unpaired two-tailed *t* test. *P < 0.05; ***P < 0.001; ns, not significant.

Apart from influenza virus, arbidol was reported to inhibit a wide array of viruses by interfering with multiple steps of the virus replication cycle^7^. The stage of SARS-CoV-2 replication targeted by arbidol was explored by conducting a preliminary time-of-addition experiment using virus at an MOI of 0.05. Arbidol was incubated with cells during the virus entry process (Entry), the post-entry stages (Post-entry), or the entire process of infection (Full-time) and progeny virus yield was quantified by qRT-PCR. The data revealed that arbidol efficiently blocked both viral entry and post-entry stages. It had a profound impact on virus Entry (~75% inhibition) with a lesser effect on Post-entry events (~55% inhibition rate) (Fig. 1b). In addition, western blot analysis (Fig. 1c) and immunofluorescence microscopy (Supplementary Fig. S2) confirmed that the expression level of viral NP was reduced drastically at Full-time (13% of the DMSO group, Fig. 1c), and showed more inhibitory effect at the Entry stage (41%) than at the Post-entry stage (61%).

The details of how arbidol blocks the entry of SARS-CoV-2 into cells were further investigated. Virus (MOI = 0.05) was allowed to bind to Vero E6 cells at 4 °C for 1 h in the presence of arbidol (10 μM) or DMSO control. Virus particles bound to the cell (bound virions) and those in the supernatant (unbound virions) were analyzed by qRT-PCR. The results showed that arbidol treatment led to a significantly decreased binding efficiency (67%) compared with the control group (*P* < 0.05) (Fig. 1d). Correspondingly, the portion of unbound virions increased significantly to 156% of the control group after arbidol treatment (*P* < 0.001) (Fig. 1d).

Next, we analyzed viral intracellular trafficking. As we reported recently, within infected cells, SARS-CoV-2 underwent vesicle transportation, which was first carried out by early endosomes (EEs) then further transported to endolysosomes (ELs)^8^. Co-localization of virions with EEs or ELs was visualized by immunofluorescence microscopy and statistically analyzed (*n* > 150 cells). As shown in Fig. 1e and Supplementary Fig. S3, in each tracked time points, there was no significant difference in the amounts of virions co-localized with EEs when comparing the DMSO- and arbidol-treated groups, although as time of infection went on (30, 60, and 90 min p.i.), the levels of co-localization considerably decreased in both DMSO- (24.0%, 5.1%, and 3.2%) and arbidol- (21.4%, 4.1%, and 2.8%) treated groups, suggesting that some virions were already transported from EEs to the next stage of vesicle transportation. By contrast, at 60 min p.i., a slightly higher percentage of virions were transported to ELs in the arbidol-treated group (22.4%) than in the DMSO group (18.3%) (*P* < 0.05) (Fig. 1e, f). At 90 min p.i., significantly fewer virions (~13.5%) were detected in ELs in the DMSO group; whereas significantly higher proportions of virions (~23.6%) remained within ELs in the arbidol-treated group, suggesting the drug trapped the virus in the ELs (*P* < 0.001) (Fig. 1e, f). Taken together, these results suggested that arbidol impeded not only viral attachment, but also release of SARS-CoV-2 from intracellular vesicles (ELs).

Among the drugs tested, laninamivir, oseltamivir, peramivir, and zanamivir are neuraminidase (NA) inhibitors, which are most widely prescribed for prophylaxis and treatment of influenza. Although no NA analog exists in SARS-CoV-2, NA inhibitors such as oseltamivir nevertheless are being used clinically in treating COVID-19 patients^1,2^. Our data showed these NA inhibitors were not active against SARS-CoV-2 (Fig. 1a), which is consistent with the finding that oseltamivir and zanamivir were ineffective in inhibiting SARS-CoV^9^. Baloxavir marboxil is a new anti-influenza drug, which selectively inhibits the endonuclease activity of the viral polymerase responsible for snatching capped primers from host mRNAs to initiate viral mRNA transcription. However, this “cap-snatching” mechanism of the endonuclease is not shared by coronaviruses that encode their own enzymes to form 5ʹ-mRNA cap structures^10^. This may explain why baloxavir failed to block SARS-CoV-2 infection (Fig. 1a). During the review process of this study, Choy et al. also showed that oseltamivir and baloxavir failed to inhibit SARS-CoV-2 in vitro^11^.

Arbidol, an indole-derivative, has been licensed for decades in Russia and China against influenza. It is a broad-spectrum drug against a wide range of enveloped and non-enveloped viruses. Arbidol interacts preferentially with aromatic amino acids, and it affects multiple stages of the virus life cycle, either by direct targeting viral proteins or virus-associated host factors^7^. For example, in influenza virus, crystal structures showed that arbidol inserted into a hydrophobic pocket of the fusion subunit of HA, thus hindering low-pH conformational change of HA and blocking the fusion process^12^. In hepatitis C virus, arbidol impaired both virus attachment and intracellular vesicle trafficking^13^. Likewise, we found arbidol plays a role in interfering SAS-CoV-2 binding (Fig. 1d) and intracellular vesicle trafficking (Fig. 1e, f). Arbidol can also bind to lipid membranes and may alter membrane configuration of the cytoplasm or the endosome, which are crucial for viral attachment and fusion^7^. It could be further investigated whether arbidol targets virus or/and cells by using published method^14^.

In summary, among the six anti-influenza drugs, only arbidol efficiently inhibited SARS-CoV-2 infection. Functionally, it appears to block virus entry by impeding viral attachment and release from the ELs. Although the SI of arbidol is relatively low (SI = 7.73), as a repurposed drug, its pharmacokinetics profile such as maximal concentration (Cmax) is more important for predicting efficacy. It is generally believed that if the Cmax achieves EC_90_, the drug is very likely to be effective; while if the Cmax achieves EC_50_, the drug is possibly effective in vivo. In humans, a single oral administration of 800 mg of arbidol results in Cmax of ~4.1 μM^15^, and this dosage is efficacious and safe against different influenza viruses with EC_50_ values ranging from 2.5–20 μM^7,16^. Arbidol also showed anti-inflammatory activity, which may enhance its efficacy in vivo^16^. Considering the EC_50_ (4.11 μM) of arbidol against SARS-CoV-2 is comparable to, or even lower than those of influenza viruses, we, therefore, suggest that arbidol is potentially effective to treat COVID-19 patients. However, the current dose of arbidol (200 mg, 3 times/day) recommended by the Chinese Guidelines may not be able to achieve an ideal therapeutic efficacy to inhibit SARS-CoV-2 infection, and should be elevated. This needs to be verified by clinical trials.

## Supplementary information

Supplementary Figs. S1-S3

## Supplementary information

**Supplementary Information** accompanies the paper at (10.1038/s41421-020-0169-8).
