# Supplementary material for: The anti-influenza virus drug, arbidol is an efficient inhibitor of SARS-CoV-2 in vitro
Source: Cell Discov. 2020 May 2;6:28. doi: 10.1038/s41421-020-0169-8 (PMC7195821; doi:10.1038/s41421-020-0169-8)
Supplement: Supplementary file 1 — Supplementary Figs. S1-S3 [file 41421_2020_169_MOESM1_ESM.pdf]

Supplementary information

The anti-influenza virus drug, Arbidol is an efficient inhibitor of SARS-CoV-2 *in vitro*

Xi Wang[1#], Ruiyuan Cao[2#], Huanyu Zhang[1, 3#], Jia Liu[1], Mingyue Xu[1, 3], Hengrui Hu[1,3], Yufeng Li[1, 3], Lei Zhao[2], Wei Li[2], Xiulian Sun[1], Xinglou Yang[1], Zhengli Shi[1], Fei Deng[1,4], Zhihong Hu[1*], Wu Zhong[2*], Manli Wang[1*]

[1]State Key Laboratory of Virology, Wuhan Institute of Virology, Center for Biosafety Mega-Science, Chinese Academy of Sciences, Wuhan 430071, P. R. China; [2]National Engineering Research Center for the Emergency Drug, Beijing Institute of Pharmacology and Toxicology, Beijing 100850, China; [3]University of the Chinese Academy of Sciences, Beijing 100049, P. R. China; [4] National Virus Resource Center, Wuhan Institute of Virology, Chinese Academy of Sciences, Wuhan 430071, P. R. China

#These authors contributed equally: Xi Wang[1#], Ruiyuan Cao[2#], Huanyu Zhang[1#]

*Corresponding authors:

Prof., Dr. Manli Wang; E-mail: wangml@wh.iov.cn

Prof., Dr. Wu Zhong; E-mail: zhongwu@bmi.ac.cn

Prof., Dr. Zhihong Hu; E-mail: huzh@wh.iov.cn

Materials and Methods

Cells, virus and antiviral drugs

Vero E6 cells (ATCC, no. 1586) were cultured in minimum Eagle's medium (MEM; Gibco Invitrogen) supplemented with 10% fetal bovine serum (FBS; Gibco Invitrogen) at 37°C in a humidified atmosphere with 5% CO_2. A clinical strain of SARS-CoV-2 (nCoV-2019BetaCoV/Wuhan/WIV04/2019) [1] was amplified in Vero E6 cells, the virus stock was titrated by an end-point dilution assay as previously described [2] and stored at -80°C. All the infection experiments were performed in a biosafety level-3 (BLS-3) laboratory.

Arbidol hydrochloride (CAS No. 131707-23-8/HY-14904), Baloxavir marboxil (CAS No. 1985606-14-1/HY-109025), Laninamivir (CAS No. 203120-17-6/HY-14818), Oseltamivir carboxylate (CAS No. 187227-45-8/HY-13318) and Zanamivir (CAS No. 139110-80-8/HY-13210) were purchased from MedChemExpress. Peramivir Trihydrate (CAS No. 1041434-82-5/S2716) was purchased from Selleck. These drugs were dissolved in dimethylsulfoxide (DMSO; Sigma Aldrich) (CAS No. 67-68-5/D2650) and were diluted to working concentration with cell culture medium. The corresponding concentration (0.25%) of DMSO diluted with cell culture medium was used as control treatments.

CCK-8 assay

The cytotoxicity of the anti-influenza virus drugs on Vero E6 Cells were determined

by a cell counting kit-8 (CCK8) (Beyotime, China) according to the manufacturer's instructions. Briefly, 1×10^4 cells were pre-seeded in 96-well plate and a series of concentrations (two fold or three fold diluted) of the compounds were added. DMSO was used as the negative control. Culture medium was set as the blank control. After drug incubation for 24 h, 20 μL CCK-8 reagent (Beyotime, China) was added and mixed thoroughly by gentle shaking, and followed by two hours incubation at 37°C. Subsequently, OD_{450} was measured in a microplate reader and the drug cytotoxicity was calculated. Cytotoxicity (%) = $1 - (A_{(drug)}-A_{(blank)})/ (A_{(negative)}-A_{(blank)}) \times 100\%$. The concentration of each drug which showed ≤ 10% cytotoxicity was chosen the starting point for evaluation of anti-SARS-CoV-2 activities. The experiments were performed in triplicate with two independent repeats and the data represent the mean ± standard deviation (SD) from two independent repeats.

Evaluation of antiviral activities

To evaluate the antiviral potential of the drugs, Vero E6 cells (1×10^5 cells/well) were cultured overnight in 48-well cell-culture plates and pre-treated with different concentrations of the compounds for 1 h at 37°C, followed by infection with virus at an multiplicities of infection (MOI) of 0.05 for 2 h. Then, the virus-drug mixture was removed, and the cells were washed with PBS and further incubated with the fresh drug-containing medium. At 48 h post infection (p.i.), the virus yield in the supernatant was quantified by qRT-PCR as described previously [2]. Briefly, 100 μL supernatant was harvested for RNA extraction with a commercial kit (MiniBEST

Viral RNA/DNA Extraction Kit; Takara, CAT No. 9766). Three microliter out of 30 μL total eluted RNA was used for cDNA synthesis with a PrimeScript RT Reagent Kit with gDNA Eraser (Takara, CAT No. RR047A). The qRT-PCR was performed with TB Green Premix Ex Taq II (Takara, CAT No. RR820A) on a StepOne Plus Real-time PCR system (AB). The primers used for qRT-PCR were RBD-qF1: 5'-CAATGGTTTAACAGGCACAGG-3' and RBD-qR1:5'-CTCAAGTGTCTGTGGATCACG-3'. The experiments were performed in triplicate with two independent repeats and the data represent the mean ± SD from two independent repeats.

Time-of-addition experiment

To determine the replication stages of SARS-CoV-2 targeted by arbidol, time-of-addition experiment was performed as previously described [2]. In all the experimental groups, Vero E6 cells (1×10^5 cells/well) were infected with SARS-CoV-2 at an MOI of 0.05 for 2 h. For "Full-time" group, cells were pretreated with Arbidol (10 μM) for 1 h prior to viral attachment. Then, the supernatant was replaced with the drug-containing medium until the end of the experiment. For "Entry" group, a procedure similar to that of the "Full-time" group was exploited, except for the cells were maintained in drug-free medium after removal of the virus inoculum. For "Post-entry" treatment, the drug was added to the cells after viral attachment.

At 16 h p.i., virus production in the culture supernatants was quantified by qRT-PCR.

The expression of viral NP in the infected cells was explored by immunofluorescence analysis (IFA) and Western blotting.

Immunofluorescence analysis

To detect the expression viral NP protein, infected Vero E6 cells were fixed with 4% (m/v, in PBS) paraformaldehyde (PFA) and permeabilized with 0.2% (v/v, in PBS) Triton X-100. After being blocked with 5% (m/v, in PBS) bovine serum albumin (BSA) at 37°C for 1 h, the cells were further incubated with the primary antibody, a rabbit serum against NP (anti-NP, 1:1000) [1], followed by incubation with the secondary antibody, Alexa 488-labeled goat anti-rabbit (1:500; Abcam). The nucleus was stained with Hoechst 33258 (Beyotime, China). The pictures were captured by a fluorescence microscopy.

Western blot analysis

Protein samples were first subjected to sodium dodecyl sulfate polyacrylamide gel electrophoresis (SDS-PAGE), and were further blotted to polyvinylidene difluoride (PVDF) membrane. After being soaked in 5% (m/v, in TBS) BSA at 37°C for 1 h, the membrane was incubated with anti-NP (1:2000) at room temperature for 2 h, followed by incubation with secondary antibody, horseradish peroxidase (HRP)-conjugated Goat Anti-Rabbit IgG (1:5000; Proteintech, China).

The mechanism of arbidol in inhibition of virus entry

To investigate the impact of arbidol on virus binding, Vero E6 cells (2×10^5 cells/well) cultured in 24-well cell-culture plates were pre-treated with arbidol (10 μM) or DMSO (0.5% v/v) for 1 h before virus attachment, and then incubated with SARS-CoV-2 (MOI = 0.05) at 4 °C to allow virus attachment for 1 h. After that, 100 μL of cell supernatant (unbound virions) was collected to determining viral RNA copy numbers by qRT-PCR as described above. The cells (bound virions) were collected after being washed three times with pre-chilled PBS, and the total cellular RNA was extracted with TRIzol Reagent (Invitrogen). One μg RNA was used for cDNA synthesis and a relative quantification was performed with viral spike gene as the target and the cellular glyceraldehyde-phosphate dehydrogenase (GAPDH) gene as an internal control, respectively. The fold change of RNA quantity in each group was calculated by the $2^{-\Delta\Delta Ct}$ method [3]. The data was normalized to the DMSO group, and P values were calculated by unpaired two-tailed t test.

The impact of arbidol on viral intracellular trafficking was investigated by co-localization analysis of virions with endosomes as previously described [4]. Briefly, Vero E6 cells (2.5×10^5 cells/well) cultured in 35-mm glass-bottom culture dishes were treated with arbidol (10 μM) or DMSO, and then infected with SARS-CoV-2 (MOI = 5) at 4°C for 1 h. The supernatant was removed and the cells were further incubated with drug-containing medium at 37°C. At different time points (30, 60, and 90 min p.i.), cells were fixed with 4% PFA. To investigate the co-localization of virions and $EEA1^+$ early endosomes, and the co-localization of virions and $LAMP1^+$

endo/lysosomes, double immunofluorescence staining of NP and EEA1 or LAMP1 was carried out as previously reported [4]. Fluorescence images were captured by a two-photon microscope (A1RMP, Nikon). The proportion of endosome (green) co-localized virus particles (yellow) to all virus particles (red) in the cells (n > 150) were quantified by image J (Colocation Threshold plugin). The statistical significance was determined by one-way analysis of variance (ANOVA).

References:

1. Zhou, P. et al. A pneumonia outbreak associated with a new coronavirus of probable bat origin. *Nature* **579**, 270-273, doi:10.1038/s41586-020-2012-7 (2020).
2. Wang, M. et al. Remdesivir and chloroquine effectively inhibit the recently emerged novel coronavirus (2019-nCoV) in vitro. *Cell Res* **30**, 269-271, doi:10.1038/s41422-020-0282-0 (2020).
3. Schmittgen, T. D. & Livak, K. J. Analyzing real-time PCR data by the comparative C(T) method. *Nat Protoc* **3**, 1101-1108, doi:10.1038/nprot.2008.73 (2008).
4. Liu, J. et al. Hydroxychloroquine, a less toxic derivative of chloroquine, is effective in inhibiting SARS-CoV-2 infection in vitro. *Cell Discov* **6**, 16, doi:10.1038/s41421-020-0156-0 (2020).

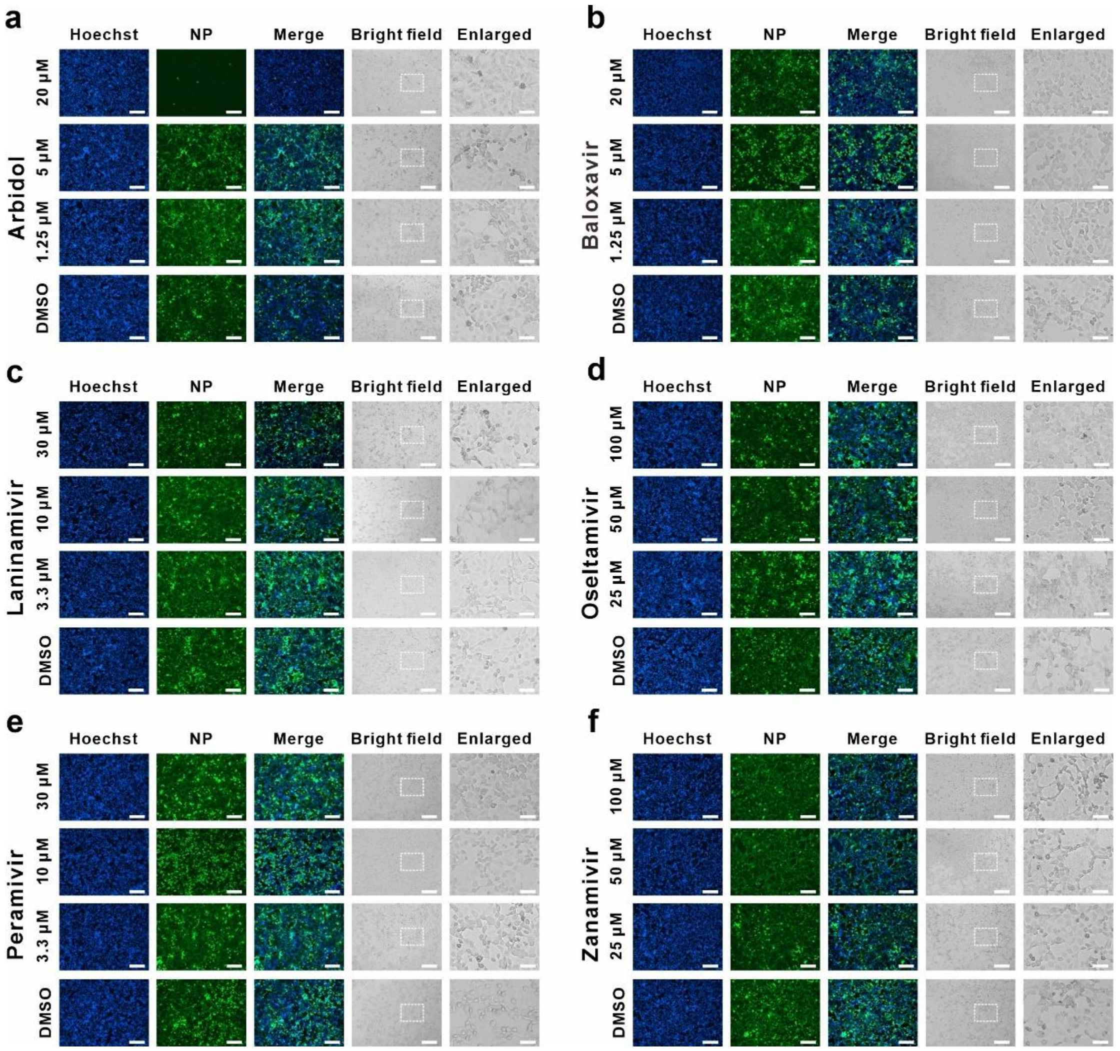

Fig. S1. Immunofluorescence microscopy of virus infection upon treatment of anti-influenza drugs. Vero E6 cells were pretreated with different doses of compounds for 1 h before infection with SARS-CoV-2 at an MOI of 0.05. Then, the supernatant was removed and the infection proceeded in the presence of drugs. At 48 h p.i., the infected cells were fixed, and incubated with anti-NP rabbit sera and stained with Alexa 488-labeled goat anti-rabbit IgG to show the expression of NP (green). The nuclei (blue) were stained with Hoechst 33258. The right panels were the enlarged images of the areas inside the white boxes in the left panels. Bars, 50 µm for the enlarged images and 200 µm for the rest photos.

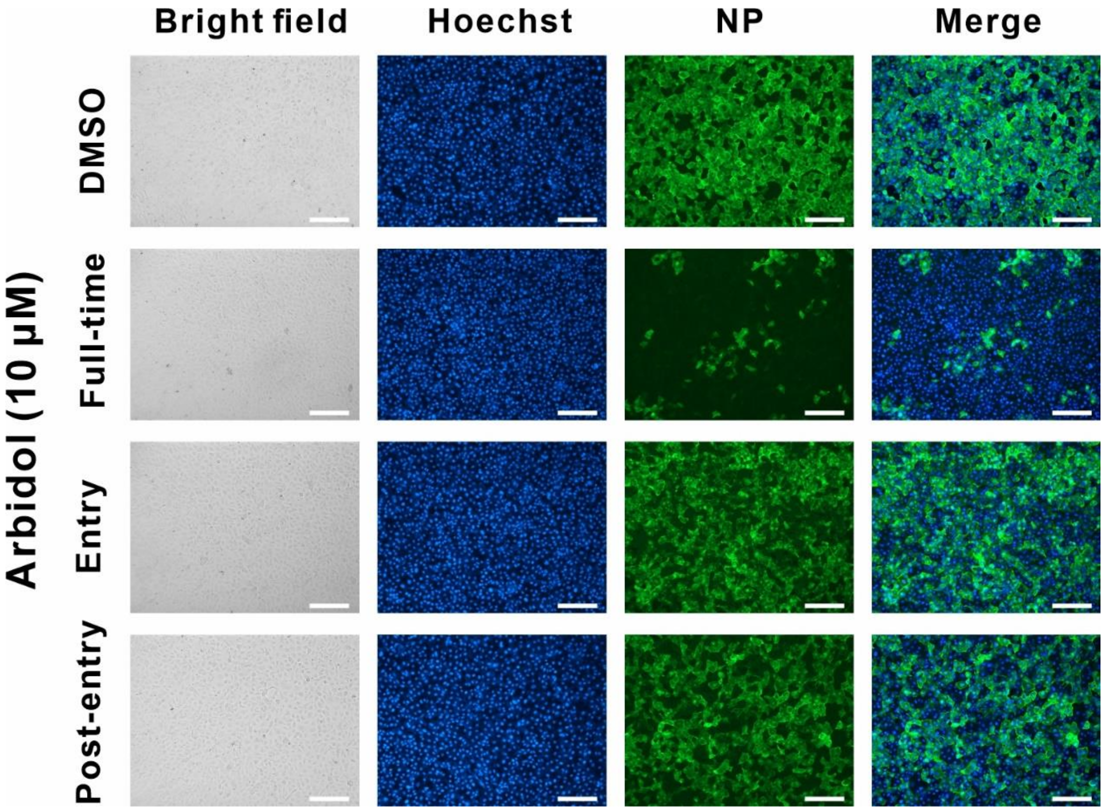

Fig. S2. Time-of-addition experiment of arbidol visualized by immunofluorescence microscopy. Time-of-addition experiment were performed as described in Materials and Methods. At 16 h p.i., the expression of viral NP in infected cells was analyzed by IFA. The anti-NP rabbit antiserum and Alexa 488-labeled goat anti-rabbit (1:500; Abcam) was used as the first and the secondary antibody, respectively. The nuclei (blue) were stained with Hoechst 33258. Bars, 200 μm.

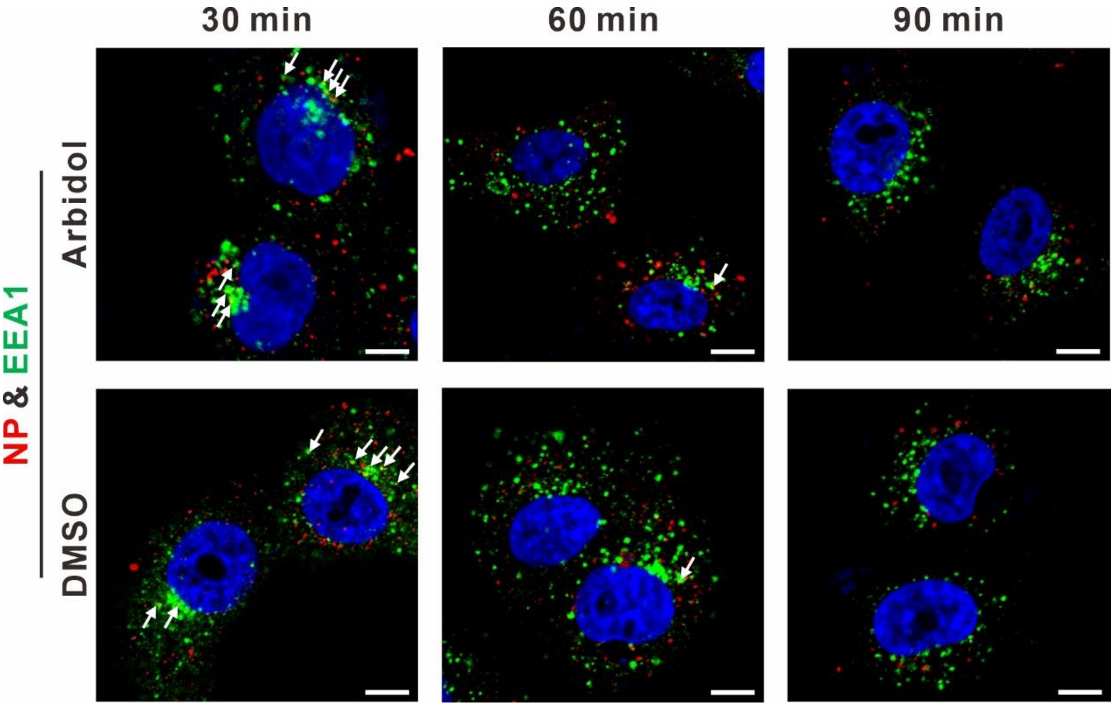

Fig. S3. Effect of arbidol on SARS-CoV-2 co-localization with early endosomes (EEs). Vero E6 cells were pre-treated with arbidol (10 μM) or DMSO for 1 h, followed by virus binding (MOI = 5) at 4°C for 1 h. After removal of virus-drug mixture, virus infection was allowed to proceed in the presence of arbidol or DMSO at 37°C for 30, 60, and 90 min before being collected for immunofluorescence assay using anti-NP polyclonal antibody for virions (red), and antibodies against EEA1 for EEs (green). The nuclei (blue) were stained with Hoechst 33258 dye. Virions co-localized with EEs in the representative confocal microscopic images of virions (red) and EEA1+ EEs (green) in each group were indicated by white arrows. Bars are 10 μm.
